# A comprehensive review on comparison among effluent treatment methods and modern methods of treatment of industrial wastewater effluent from different sources

**DOI:** 10.1007/s13201-022-01594-7

**Published:** 2022-03-21

**Authors:** K. Sathya, K. Nagarajan, G. Carlin Geor Malar, S. Rajalakshmi, P. Raja Lakshmi

**Affiliations:** 1 Department of Biotechnology, Rajalakshmi Engineering College, Thandalam, India; 2 Department of Chemical Engineering, Rajalakshmi Engineering College, Thandalam, India

**Keywords:** Industrial growth, Environmental degradation, Resource recovery, Challenges and perspectives, Recent developments, Hybrid/integrated system

## Abstract

In recent years, rapid development in the industrial sector has offered console to the people but at the same time, generates numerous amounts of effluent composed of toxic elements like nitrogen, phosphorus, hydrocarbons, and heavy metals that influences the environment and mankind hazardously. While the technological advancements are made in industrial effluent treatment, there arising stretch in the techniques directing on hybrid system that are effective in resource recovery from effluent in an economical, less time consuming and viable manner. The key objective of this article is to study, propose and deliberate the process and products obtained from different industries and the quantity of effluents produced, and the most advanced and ultra-modern theoretical and scientific improvements in treatment methods to remove those dissolved matter and toxic substances and also the challenges and perspectives in these developments. The findings of this review appraise new eco-friendly technologies, provide intuition into the efficiency in contaminants removal and aids in interpreting degradation mechanism of toxic elements by various treatment assemblages.

## Introduction

Globally, the growth of any country can be absolute based on its industrial growth. Industrial sectors can be of various types based on the products. Rapid industrialization and its concentration in or near urban centers have placed very high pressures on the carrying capacity of the environment at specific locations. At these locations, water bodies such as rivers, lakes, and coastal waters have typically been severely affected by the release of the contaminants into them. Industrial wastewaters are effluents that result from human activities which are associated with raw-material processing and manufacturing. These wastewater streams arise from washing, cooking, cooling, heating, extraction, reaction by-products, separation, and quality control resulting in product rejection. Older method of treatment, which is built with the centralized point of view, is showing less effectiveness which ultimately leads to the ever-increasing accumulation of the effluents. With the development of new methods has reduced the hardships caused by the older ones. Wastewater treatment plants are developed to improve the water quality significantly to meet the safety requirements of the effluent after treatment. Various treatment methods reduce the concentration of pollutants in water and also eliminate the content of suspended solids, whose molecules can contaminate the rivers and impede the movement of water in the channels and pipes after deposition. It also degrades the content of biodegradable organic matter, measured by the Biological Oxygen Demand (BOD).

Wastewater treatment is required as a part for reducing contaminants to a sufficient degree to obtain potable water. Therefore, the treatment plant is to be designed in such a way that it takes into consideration some parameters in the influent that is required to be controlled to enhance the efficiency of the treatment plant. Due to uncontrolled entry of wastewater effluent into the environment and the transportation of contaminants into anthropoid system, environmental protection requires the use of suitable purification/treatment systems with high removal efficiency for contaminants are needed. Economically, effective wastewater treatment has important effects on saving water, and preventing unnecessary water scarcity. This study focuses on summarizing the treatment methods of effluent generated by various industries and its efficacy in removing the pollutants, taking into account the characteristics of wastewater and geographical location of the relevant industries.

## Industrial growth and its impact

Industrial development plays a key role in economic growth of a country. Industrial evolution is obligatory for rejuvenation of agriculture since chemical fertilizers, pesticides, weedicides etc. are all industrial products that are vital to increase the productivity and also, it uplifts the progress of science and technology. Critical lack of capital is the foremost issue of Indian economy. With the assistance of apparent and interior wealth, industry can acquire greater profit that can be revived for growth and development. Industrialization aids in the advancement of trade. Concerning international trade, import substitute product production and export promotion are necessary to meet the deficit in balance of payments that are achieved through industrial development. The production and consumption of electricity lead to environmental impacts which must be considered in making decisions on the way in which to develop energy systems and energy policy. All forms of electricity generation, and indeed all parts of the fuel chain that have impacts, both positive and negative. In the decision-making aspect of the process, consideration of the real energy needs of the country and the values of the society must be taken into account.

### Statistical inference on global industrial growth:

**Fig. 1:**
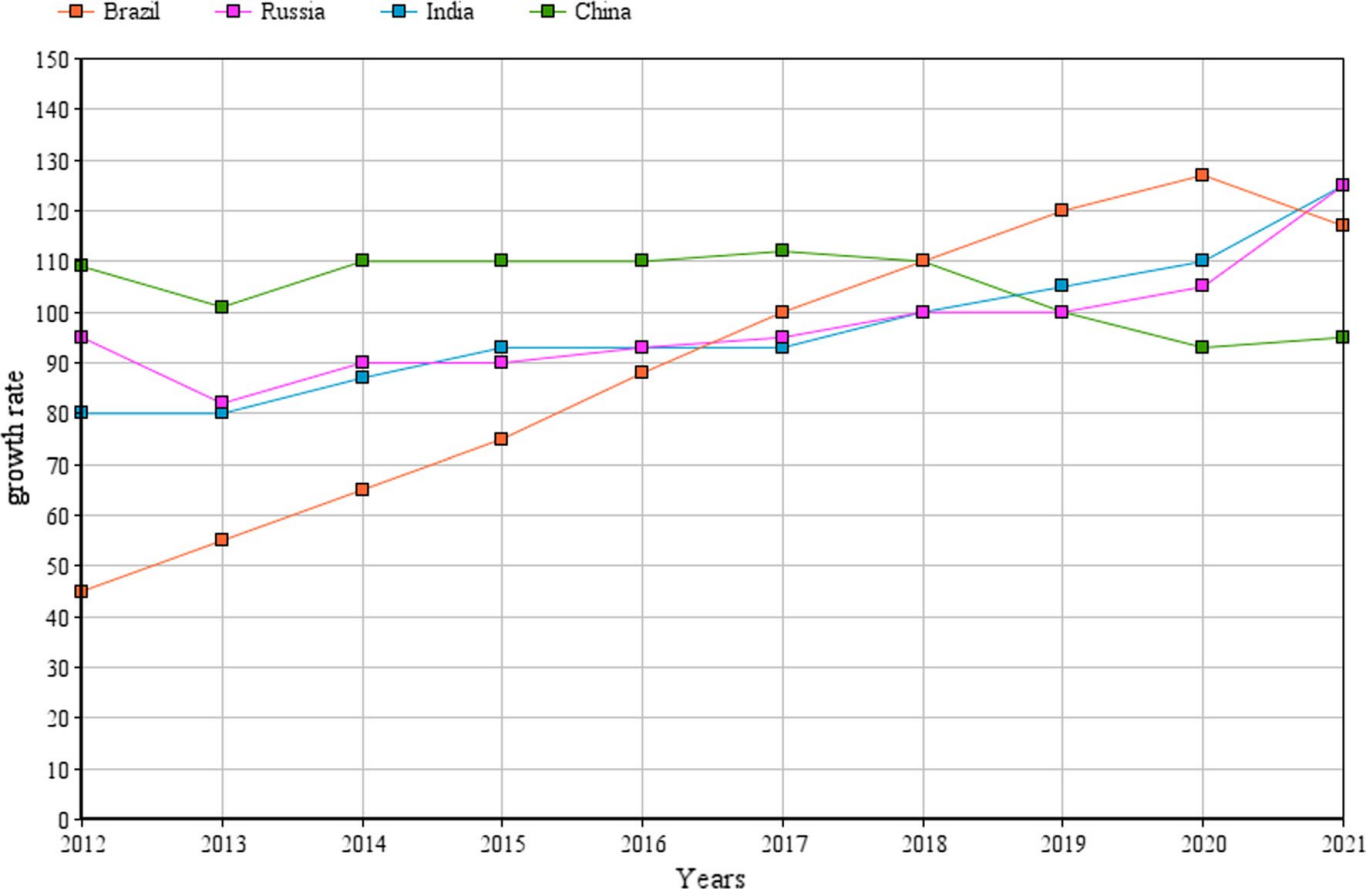
Statistical data on global industrial growth (Radulescu IG et al. 2021, Christina Majaski. 2020, Svetlana Gusarova. 2019, Rachel S Salzman. 2021) (Standard of Fig. 1 is improved including caption)

Figure 1 is the graphical representation of industrial widening of BRIC countries in recent decade (2012–2021) which interprets that there are several industries that conceals these countries with reference to their imports which is unable to give over in terms of trade development. The key finding is that life science industrial products ranks high marketing rate (~ 70%) and at the same time, generating greater amounts of toxic effluents (Devi Prasad Dash et al. 2021). COVID-19 influences the industrial broadening of BRIC countries that leads to 50% reduction in their growth, however, yet shows superior global average. The worldwide industrial production is intensified by the reports that quarterly explored the circumstances of the global industrial development and its outlook. This outline deals with structural changes in the course of industrial growth such as:

- Brief series of economic crisis since early 2000s
- Extensive crisis
- Prolonged crisis overcoming
- Assailing national liability
- Stagnation and setback of industrial growth leading to a static total propensity.

And also, the time from the outbreak of the world economic crisis and depicts the essentially changed process of the crisis overcoming. Industrial production in the MIST (Mexico, Indonesia, South Korea, Turkey) countries with a 6% share of machinery exports are evolving after stagnation and decline in the first quarter of 2020 and has its basis in the period after the global economic crisis. China’s industrial production increases by 9% yearly and those of India by 3.4% and Russia by 2% per annum. In the previous global economic crisis of 2008, China had bit alleviated the slump in industrial growth in industrialized countries (Avunduk et al. 2019). On the other hand, no OECD (Organization for Economic Co-operation and development) data are available for Brazil and India for the first quarter of 2020.

### Critical environmental impacts

Unconstrained industrial wastewater is the probable origin of spontaneous release of hazardous substances into the environment. It has been identified that wastewater generated by various industries leads to health effects such as cancer, immune function disorders, lung and respiratory diseases (Upadhaya et al. 2017). State Inspectorate for Environment Protection (PIOS) reported that, about 60% of wastewater creates potential or actual threat to public health and the environment. For safer and reliable wastewater management, risk assessments such as hazard identification, exposure assessment and risk characterization are to be considered (Buczyńska et al. 1999).Generally, industrial effluent carries certain disease-causing bacteria such as E.coli, Salmonella etc., that leads to cholera, typhoid fever and other allergic symptoms (Tiffon C et al. 2018).

In recent years, there have been the development of a disease namely byssinosis, is a fatal disease caused to the people working in textile industry on account of excessive exposure to cotton dust. Myeloid leukemia, a disease majorly caused to workers in organic chemical industry due to their over exposure of formaldehyde without much safety measures taken in prior (Gaur VK et al. 2020). “Asthma-like syndrome” is a nonallergic respiratory condition that is identical clinically to asthma but is not associated with persistent airway inflammation or airway hyper reactivity. As the pulmonary deterioration can often be detected only by cross-shift testing, it can be difficult to document this in a typical clinic setting (Etim MA et al. 2021). The cross-shift decline in FEV1 is generally less than 10% but can be between 10 and 15%. It is most common in swine confinement workers, up to 10% acutely, but can also be seen in grain workers of agricultural industry.

## Extensive sources of industrial effluent

### Electric power plants

Electric power plants are one of the largest industrial sectors that is involved in meeting the electricity requirements throughout the world. There are few major unit processes involved in electric power plants. First, the fuel materials are transported to the power plants and are pulverized to increase the efficiency and stored. Then, the fuel is burnt and the heat produced is sent to boilers that in turn changes the liquid into steam (Srikanth 2015). The obtained steam is then allowed to pass through the turbine where the turbine starts to rotate and produces electricity. Finally, the steam is passed through the condenser which condenses the outlet steam from the steam turbine in the form of pure water. The water is then pumped back to boiler.

**Fig. 2:**
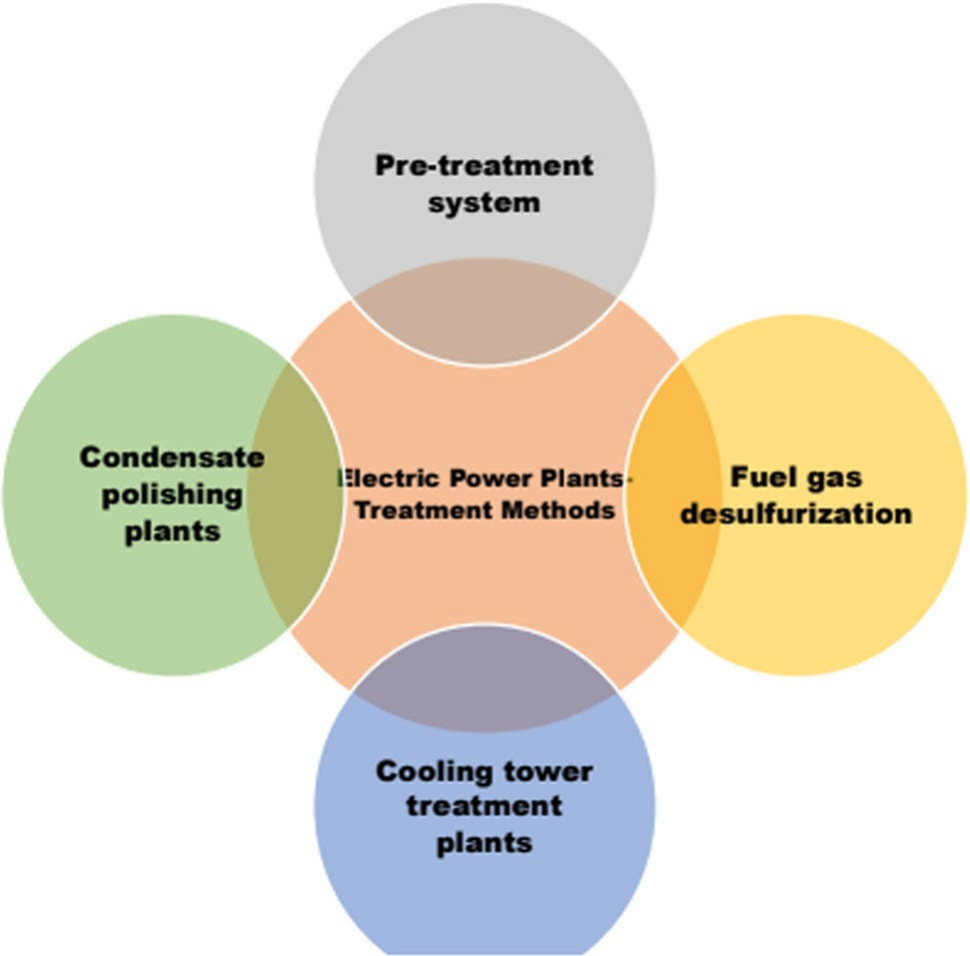
Treatment methods of electric power plants effluents

Amongst the above-mentioned stages, condensation of the steam produces waste water effluents. Though wastewater effluent parameters do not exceed the sewer system limit, they exceed irrigation standards that are of concern. The methods involved to treat the effluents from electric power plants are depicted in Fig. 2 and the studies that reported on parameters involved in effluent analysis of electric power plants are detailed in Table 1.

**Table 1:** Waste water effluent analyses of the electric power plants (data provided in this table has been updated)

Parameters	Average	Maximum	Standard sewer	Standard irrigation	References
Temperature (°C)	29	36	–	–	Ravindra.D. Kali et al. (2018)
_P_H	7.9	9.4	5.5–9.5	6.5–8.4	Ravindra.D. Kali et al. (2018)
BOD (mg L^−1^)	13	43	800	–	Ravindra.D. Kali et al. (2018)
COD (mg L^−1^)	28	64	2100		Prerana kane et al. (2016)
Zn (mg L^−1^)	0.3	6.75	15		Prerana kane et al. (2016)
Cl (mg L^−1^)	–	711	–	350	Saha PD et al. (2015)
FOG (mg L^−1^)	0.5	2.0	50	5	Saha PD et al. (2015)

*BOD* biological oxygen demand; *COD* chemical oxygen demand; *FOG* fats, oils and grease

### Battery manufacturing industry

Battery industry is represented as one of the most important toxic and hazardous industry. Generally, the batteries are made up of a positive electrode, a negative electrode and an electrolytic solution. The manufacturing process involves oxide milling, grid casting, paste manufacturing, grid pasting, plate curing, plate parting and battery assembly (Rahangdale et al. 2012).

The manufacturing industry uses water for the preparing reactive materials and electrolytes, for depositing reactive materials in the surface. As a consequence, wastewater is generated and is characterized based on the high levels of cadmium, Nickel, silver and also depends on the process adapted in battery making.

### Nuclear power plants

The Nuclear power plants produce radioactive substances as effluents which are released in airborne and liquid forms. Airborne effluents are released mainly by nuclear fission and activation of gases like tritium. Both Pressurized Water Reactor (PWR) and Boiler Water Reactor (BWR) release these effluents (Efremenkov VM 2014). The instability in airborne effluent is based on designs and operations of radioactive waste management, effluent control systems and analytical methods that are employed to monitor the effluent.

**Fig. 3:**
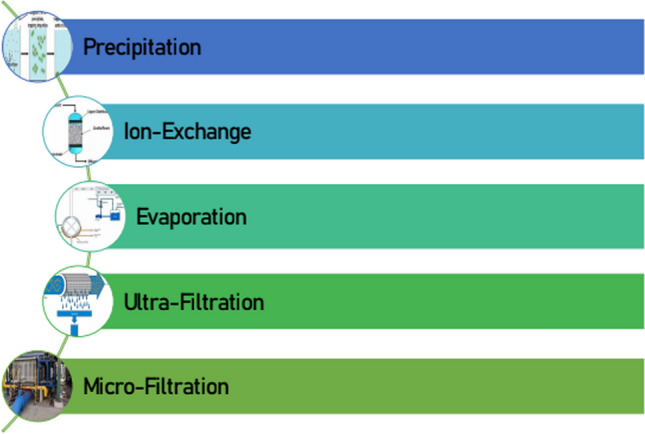
Methods in reduction of nuclear liquid effluents

Liquid radioactive effluents that are released in surface water are monitored prior to authorize release to the sea. In addition, uncontrolled leaks of liquid radioactive effluents due to fission activities have resulted in contamination of groundwater. Tritium activity in liquid effluents is much higher for PWR than BWR. Currently, nuclear plants typically release a few curies of tritium in liquid effluents due to the mixed fission and activation products. The method available for the reduction of Liquid effluents produced by the nuclear power plants is illustrated in Fig. 3. Table 2 shows various commonly reported categorized radionuclides.

**Table 2:** Common radioactive nucleotides

Category	Commonly reported radionuclides	References
Fusion and Activation gases	Krypton, Xenon, Argon	Stram et al. (2015)
Halogens	Iodine, Bromine	Harris JT et al. (2011)
Particulates	Cobalt, Cesium, Chromium, Manganese, Niobium	Harris JT et al. (2011)
Mixed fission and activation products	Iron, Cobalt, Cesium, Chromium, Manganese, Zirconium, Niobium, Iodine	Kamdi et al. (2013)
Tritium	Hydrogen	Kamdi et al. (2013)
Dissolved and entrained noble gases	Krypton, Xenon	Stram et al. (2015)

### Mines and quarries

Mining generally refers to the process of removing of rocks, sand and other minerals from the ground. The place where the mining takes place is called as quarries. This industry generally pollutes all the form of natural resources that surrounds them (Kuyucak et al. 2008). Majorly, the waste water released from mines and quarries are highly toxic in nature. These highly toxic metals influence the water bodies and leads to several diseases. The effluents released are treated in different process that includes Neutralization, Chemical oxidation, Biological Treatment and Co-precipitation.

### Petroleum industries and petrochemicals

Petroleum industries produce both liquid and gaseous pollutants. Gaseous pollutants are easier to manage compared to liquid pollutants. Effluent Treatment Plant (ETP) can efficiently treat the liquid pollutants to protect the environmental pollution (Saha PD et al. 2015). The treatment systems for the effluents produced by the petroleum industries (Table 3) involves removal of coarse suspended and floating matters, oil, grease, organic solids through biological processes, colloidal particles and refractory organics. The oil–water separation method is widely practiced in American Petroleum Institute (API) to separate oil and grease.

**Table 3:** Literature on petroleum waste water treatment, parameters and treatments

Pollutant type	Parameters	Treatment	References
Petroleum refinery effluent	COD, BOD, Oil and Grease	Identified photocatalytic degradation as efficient technique	Henrik Pederson et al. (2014)
Oil and greasy effluent	COD	Significant amount of COD (about 85%) reduction within 10 min	Correia T et al. (2015)
Heavy oil produced as effluent	BOD, COD, TKN, Oil	Surface flow constructed wetland can be removed	Correia T et al. (2015)
Highly saline waste water	NaCl, turbidity	RO is efficient for removing the salt concentration and organic matters	Mishra A et al. (2011)
Oily waste water	COD and oil Content	Photocatalytic decomposition of oily waste water by modification of catalyst activity	Henrik Pederson et al. (2014)

### Organic chemical-manufacturing industries

Amongst all the industries, the chemical industries impose a great impact on the environment. The wastewater released from this industry is generally highly concentrated with organic and inorganic pollutants which may be toxic (Fayza NA et al. 2007). Most of the effluents released are observed to possess mutagenic, carcinogenic, and non-biodegradable properties. Most common methods are used for the treatment of chemical effluents such as dissolved air floatation, de-emulsification, gravity separation, skimming, coagulation and flocculation.

### Iron and steel industry

In iron and steel industry, wastewater generated from coke oven by-product plant is considered to be the most polluting. This wastewater contains toxic chemicals like phenol, cyanide, and ammonia which are harmful to the receiving water bodies. Various harmful effects of the untreated wastewater from steel industry are reported to be toxicity to aquatic life, reduction of Dissolved Oxygen (DO) silting due to suspended solids (Ammonia and phenol released in the effluent increases pH of the water and thus is responsible for the toxicity. Due to the discharge of biodegradable organic substances into the water bodies, the soil and bacteria use the organic matter as carbon source and cause reduction in the DO level in the water. Hazardous effects caused by the effluents of this industry are listed in Table 4.

**Table 4:** Toxic effects caused by various pollutants generated from effluent of iron and steel plant

Class of pollutants	Toxic effect	Disinfection	Reference
Heavy metals	Poisonous interference to enzyme systems and metabolism of body	Blood and cardiovascular, reproductive and urinary system	Sinha S et al. (2016)
Aromatic compounds	PAH have different type of toxic action, depending on the compound	Non-polar narcosis, photo toxicity results in mutagenicity and Carcinogenicity	CL Beh et al. (2014)
Surfactants	Enhance the bio availability and stimulate the biodegradation	Negative impact on the survival of heterotrophic nanoflagellates and ciliates	Das et al. (2018)
Cyanides	Dynamic effects depend on the dose, route and speed of administration including the physical condition of Recipient	Lethal toxicity after inhalation of hydrogen cyanide gas affects many functions in the body	Abhay et al. (2018)
Fluorides	Intake of 20–40 mg/day can inhibit the important enzyme phosphatase	Osteoporosis and arthritis, cancer, infertility brain damage	Pallabi Das et al. (2018)

Entrapped oil and grease from the effluent lead to the formation of slicks and poor aesthetics. In the existing plants, Coke Oven by Product (COBP) wastewater is treated by biochemical oxidation of cyanide, ammonia and phenol. These effluent treatment plants are commonly known as Biochemical Oxygen Demand (BOD) plants. Different oxidation techniques had been implemented to reduce pollution, mainly caused by organic compounds.

### Food industry

**Fig. 4:**
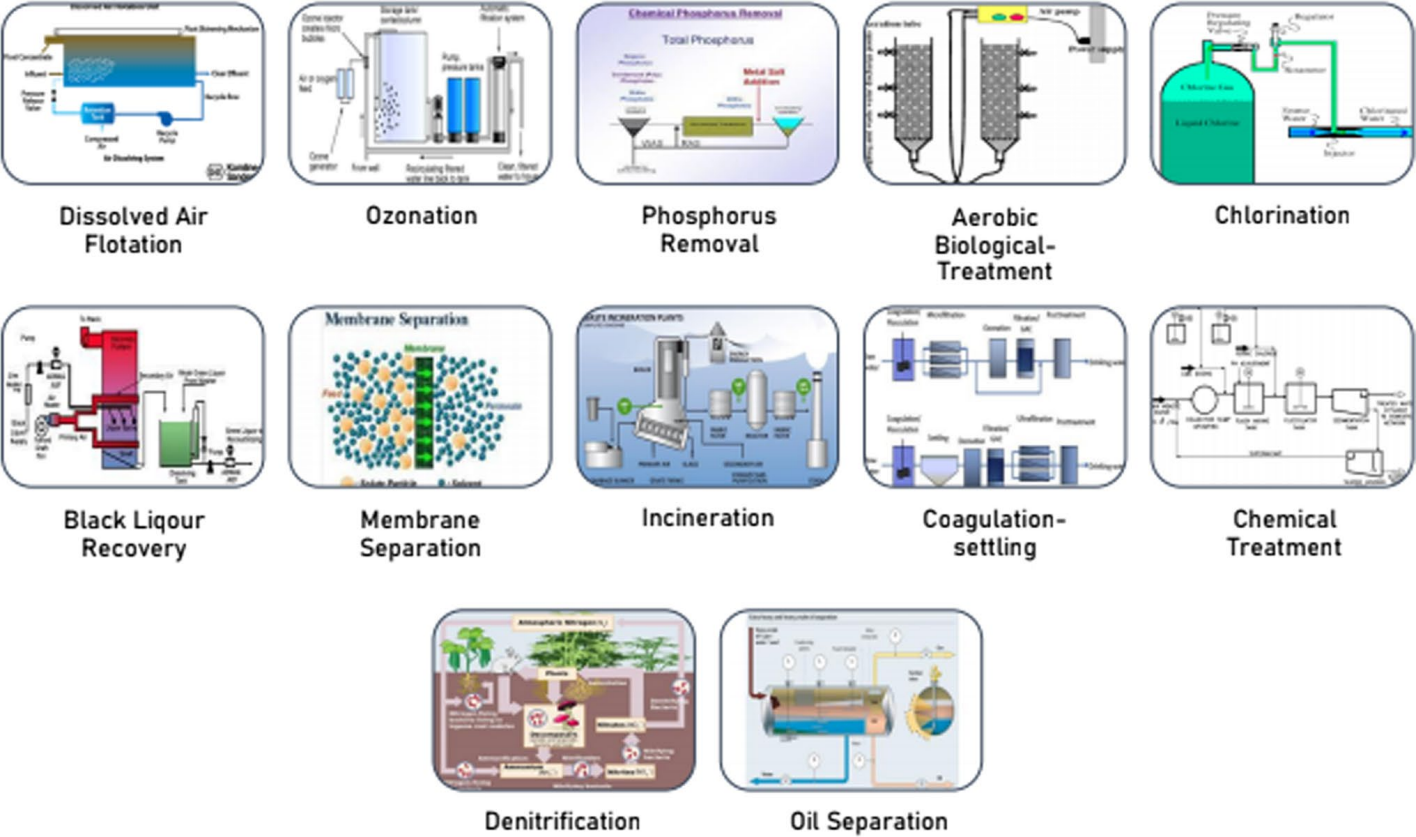
Various treatment methods in food industry

The waste water released from the food industry causes environmental pollution due to its high Chemical Oxygen Demand (COD) and BOD content. In comparison with other industries, food industry requires great amount of water, since it is used throughout most of plant operations. Noukeu NA et al. (2016) reported the variation in BOD/COD, total solids and suspended solids in the effluents of these industries. This is due to the different additives used for various food products. Chocolate industry is among the most polluting of the food industries with regards to its large water consumption. Different methods of effluent treatment in food industry are depicted in Fig. 4.

### Dairy industry

Generally, the waste water generated from the dairy industry contains high amount of solid organic compounds. The dairy industry mainly handles large quantity of milk that contains large quantities of milk constituents like casein, inorganic salts (Raghunath BV et al. 2016). The quality of the effluent is majorly determined by quantity of milk processed and the type of product manufactured. Characteristics of dairy effluent are illustrated in Table 5. The effluent treatment in dairy industry is majorly done by the physicochemical methods like chemical precipitation, coagulation/flocculation, membrane process, etc., which have up to 98% efficiency.

**Table 5:** Parameters in dairy industry effluent (data provided in this table has been updated)

Parameters	Effluents released	Average	References
P^H^	1–5.8	6.5–8.0	Shete BS et al. (2013)
Temp (°C)	25–50	33–38	Zhang T et al. (2018)
BOD (mg L^−1^)	1300	100	Kolhe AS et al. (2009)
COD (mg L^−1^)	2400	250	Ahmed T et al. (2019)
TSS (mg L^−1^)	700	100	Sinha S et al. (2019)
TDS (mg L^−1^)	5600	2100	Tikariha A et al. (2014)
Oil & Grease (mg L^−1^)	35	10	Ahmed T et al. (2019)

### Oil extractive mills

Edible oil is one of largest used product in households. These oils are manufactured in mills where they undergo different process like cleaning the seeds, grinding, pressing, extraction of oil, bleaching, deodorization, pre-chilling and packing. Mostly, the wastewater is generated from the bleaching process and the ETP unit (Rajkumar K et al. 2010). The effluents undergoes primary treatment and undergoes secondary process like physicochemical (Air Flotation, Coagulation, Flocculation) and biological process.

### Leather industry

Leather industry is one of the fastest growing and most polluting industries globally. The leather processing is responsible for the unfavorable conditions of environment. The leather obtained by processing is used for various purposes like shoes, textile, carry bags and many more. For this, huge numbers of raw materials is collected and are subjected to chemicals for obtaining the complete finished product (R Belaabed et al. 2014).

Generally, the skin of the animals is mainly composed of water, protein and fatty materials. Leather manufacturing undergoes stages such as: preparatory, tanning and crusting. The unwanted raw skin and hairs are removed through soaking, unhairing, liming, de-liming and bathing. During tanning process, unstable raw materials are converted to leather with adequate strength properties and resistance to biological and physical attacking agents (Mirulanini V et al. 2017). Most commonly used tanning agent is chromium (Cr(III)) salt that is added after pickling to increases the pH of the hide and to obtain leather of higher thermal and bacterial resistance. The chromium tanned leather contains about 2–3% of dry weight of chromium salts.

The wet processing includes re-tanning with chromium and also addition of some dye and fats for improving the smoothness and color and after several process of drying and re-tanning, the product is available in the form of crusts. The crusts obtained is subjected to undergo the finishing process (Tapan Biswas et al. 2013). Finishing process is undergone to obtain softer and clean leather as product. The finished end product has about 65–80% of the dry weight of chemicals used. In leather industries, water is used mainly in the tanning process. In addition to the usage of water in every individual step, water is also used in vacuum dryer.

### Pharmaceutical industry

Pharmaceutical is one of the major industries that pollute the water bodies in large amount as they use about 99% of water for the production of excipients. The water released from this industry is basically from drug manufacturing area that consists of various toxic elements that are harmful for both human and animals. Various treatment methods like membrane filtration, reverse osmosis, flocculation and coagulation are applied to reduce the presence of elements like BOD, COD, TSS, TDS from the effluent (Azizi E et al. 2017).

### Agriculture industry

Commercial agriculture industry consumes large volume of water from different sources and generates a huge amount of waste water. These wastewaters generally consist of pollutants like organic matters, inorganic matters (dissolved minerals), Nutrients (Nitrogen, Phosphorous, Potassium), Toxic chemicals, Pathogens (Levy GJ et al. 2011). Various filtration processes involved will remove fertilizers, Manure, Suspended solids, Virus & Bacteria, BOD, COD, Pesticides.

The effluent treatment in agriculture industry involves in mechanical methods like filtration, sedimentation, separation, flotation, crystallization or in biological methods like activated sludge at anaerobic conditions and also in physicochemical methods like electro-coagulation, coagulation and flocculation, ozonation (Oron G et al. 2012).

### Textile industry

Textile industry is one of the largest and oldest industry presents in the world. Out of various processes in textile industry, contribution of chemical processing is about 70% of the pollution (Desai PA et al. 2011).

The effluent treatment in textile industry involves the removal of suspended solids and to reduce the quality of effluent using methods like screening, sedimentation, neutralization, Mechanical flocculation and chemical coagulation (Khandegar V et al. 2013). The secondary treatment mainly involves the removal of colloidal and dissolved organic compounds and also for the removal of dyes/color present in the waste water which is usually done using aerated lagoon, anaerobic digestion, trickling filtration and then the tertiary treatment is usually done to increase the efficiency of the little contaminated water obtained from the above process to release into waterbodies without any problems to the waterbodies which is obtained by various methods like oxidation technique, Electrolytic precipitation, membrane technologies.

### Paper and pulp industry

Paper and pulp possess a huge impact on our everyday life. The paper and pulp industries produce huge quantities of biomass (Hubbe et al. 2016). The production of pulp and paper involves many steps such as Debarking, Chipping, Pulping, Bleaching and various studies on the variants of pulp and process parameters are described in Table 6.

**Table 6:** Composition of effluent from paper and pulp industry (Data provided in this table has been updated)

Process	BOD (kg/L)	TSS kg/L	COD (kg/L)	Pt-Cd (kg)	References
TMP	13–22		50–80		Gautam et al. (2016)
Mechanical	8.5–14	10–30	20–55		Gautam et al. (2016)
Bisulphite				10–30	Sudarshan et al. (2017)
Unbleached	25–50	10–110		75–150	Cabrera et al. (2016)
Bleached	20–60	20–150	35–120		Cabrera et al. (2016)
Kraft-unbleached	1–20	0.2–15	7–50	20–50	Elliot et al. (2017)
Bleached normally	0.2–40	0.2–10	4–90	100,240	Elliot et al. (2017)
OZP bleaching	1–20			40–80	Cadat et al. (2011)
CTMP	17–30		60–100		Forster et al. (2009)
Bleached	20–40	10–30	80–130		Forster et al. (2009)

The effluent treatment in paper and pulp industry usually involves primary treatment methods like sedimentation, flotation, filtration and the Secondary treatment (biological) such as activated sludge, aerated lagoons, anaerobic treatment (Hydrolysis, Acidogenesis, Acetogenesis, Methanogenesis) and sequential treatment. Tertiary treatment involves membrane separation (Microfiltration, Ultrafiltration, Nanofiltration, and Reverse Osmosis), coagulation/precipitation, and ozonation (Table 7).

**Table 7:** Types of pulp and their uses

Type	Variant	Description	End-use	References
Mechanical pulp	Stone Ground wood Pulp	Mechanical grinding of the wood into short fibers	Used in the newsprint and wood containing paper, such as lightweight coated Papers	Devendra singh et al. (2017)
	Thermo- mechanical Pulp (TMP)	The wood particles are softened by the steam before entering the pressurized refiner	Mainly used in the super calenderer papers	
Semi-chemical pulp	Semi-Chemical Pulp	Produced in the same way as of TMP, but the wooden particles are made to undergo the chemical treatment before entering the refiner	Used in the tissue manufacture. Some CTMP is also used for writing and printing Grades	Omid Ashrif et al. (2018)
Chemical pulp	Sulphite Pulp	Produced by cooking wood chips in the pressure vessel in the presence of bisulphate liquor	Varies from newsprint, printing and writing Papers	Cadet et al. (2016)
	Sulphate/Kraft	Pulp is produced by cooking the chips in the pressure vessel in the presence of sodium hydroxide liquor	Pulp used for the graphic designing papers tissues and carton boards	

## Industrial wastewater and reserves rehabilitation

Issues such as water shortage, nutrient needs, and low fossil energy consumption could be conquered by implementing resource recovery technologies. Various products can be recovered from wastewater apart from nutrient, energy and reusable water. There are wide range of recovery strategies available that sustain that includes chemical production, raw commodity chemicals, energy recovery, fertilizers, animal feeds and consumer products (Silva B et al. 2021). Techniques like partition-based biological process, acid-bioleaching, thermo- and meso-anaerobic digestion, bio-precipitation using bioelectrical system, trans-esterification (separation) are in practice for the effective recovery of valuable products such as biochar, single-cell protein-based foods, bioplastic, struvite (MAP—Magnesium, Ammonia, Phosphate compound), biogas, metals, radionuclides, etc. (Hussain MI et al. 2019). Resource recovery and reuse (RRR) strategy is being broadly implemented in wastewater treatment plants for purification and reuse of resources.

Water reclamation and reuse technologies (desalination/long-distance fresh water transfers) are considered to be a more feasible choice driven by water scarcity due to unbalanced global fresh water administration and water stress due to climate change. Advanced treatment lines are required to encounter the firm permissible quality for microbes and micro-pollutant concentrations in reclaimed water, since water retrieved after secondary treatment contains residual concentration of organic micro-pollutants like polychlorinated biophenyls, pesticides, pharmaceuticals, etc. Technologies such as: (Meeroff DE et al. 2019).

- Filtration
  o Membrane-based: micro-, ultra-, nanofiltration and reverse osmosis
  o Non-membrane: sand and biochar filtration, trickling filter, activated carbon (granular/biological)
- Disinfection: chlorination, UV radiation
- Advanced oxidation process: ozonation, Photocatalytic oxidation, cavitation, use of fenton/H_2_O_2_

are employed to reclaim water from wastewater treatment plants. Table 8 represents technologies for the recovery of valuable substances from different industrial wastewater.

**Table 8:** Technologies for the recovery of valuable substances from different industrial wastewater

S.No	Industry sectors	Valuable products recovered		Technical method applicable for recovery	Advantages	Disadvantages	References
		Recovery of valuables (metals, solvents)	Recovery of process streams (electrolytes, inorganic acids)				
1	Electrical power plants	Non-ferrous metals like copper, zinc, tin etc. and	Cr, Zn—electrolytes,	Chemical oxidation, reduction	High reaction rates, provides complete mineralization of organic compounds	Operational problems as other reference electrodes are used	Liu M et al. (2018)
2	Nuclear power plants	Uranyl nitrate (for the conversion of uranium to fuel)—using tributyl-phosphate	Boric acid, polyantimonic acid, hydrous titanium oxide (inorganic sorbents used for treatment of radioactive waste streams)	Selective ion exchange	Selective removal of specific radionuclides, low cost, no addition of chemicals	Large p^H^ changes in the production process, time consuming	Ohto H et al. (2017)
3	Mining	Gold, silver, copper, nickel, niobium, tantalum, cobalt, zinc, zirconium & other rare earth elements	Glutaric acid (from leaching process), H_2_SO_4_	Extraction and using special adsorbents	Highly effective process with rapid kinetics	Expensive	Mfune O et al. (2018)
4	Ceramics	Tantalum, niobium oxide—using liquid membranes	Solvents like xylene present in acrylates, epoxies, etc.	Cementation	Controlled potential permits for the separation of precious metals, effective when carried out by reduction with metallic iron	Excess conciliatory metal consumption	Jouhara H et al. (2021)
5	Pharmaceutics	Acetone, hexane, isopropanol	Electrolytes like sodium chloride, calcium gluconate	Chromatography	High accuracy, precision, recovery	Since the eluent is itself an electrolyte, it is difficult to determine the separated analytes against eluent	Savelski MJ et al. (2017)
6	Food	Deep eutectic solvents like choline chloride with glycerol, phenyl acetic acid that is used for the separation of organic compounds such as phenolic, aromatic, sugars, flavonoids from food samples	Nitric acid (mineral acid), hydrochloric acid, lactic acid, etc.	Crystallization and evaporation	Low temperature and less energy requirement	Yield is limited by phase equilibrium	Hernández K et al. (2021)
7	Machinery	Oils	Acetone, hexane, xylene, methyl ethyl ketones, alcohols	Resin adsorption	High capacity and selectivity of the resin	Excess rinse time and migration of cation resin into anion unit can cause leakage problems	Dutournié P et al. (2019)
8	Organic chemicals	Organic solvents like acetone, isopropanol, methanol, methanol, ethanol, hexane	Inorganic acids such as HCl, HNO_3_, H_2_SO_4_	Acid and ion retardation	High accuracy, recovery and regeneration limits the emission of harmful gases	High energy consumption, lack of selectivity towards heavy metals	Wang S et al. (2019)
9	Agriculture	Special metals like lead, chromium, arsenic, zinc, cadmium, copper, nickel, etc.	Sodium sulfate, hydrofluoric acid (applied in the production of insecticide and fertilizer)	Sulfide and organosulfate precipitation	Highly efficient towards heavy metals and feasible	Formation of oligomers	Xu M et al. (2021)
10	Battery manufacture	Metals and metal-oxide such as nickel, lithium, cobalt-oxide	Electrolytes like NaCl, KCl	Hydroxide-precipitation	Low cost of execution, simple process, easy p^H^ adjustments	Low solubility of the metal, sensitive to the concentration of precipitating agent	Chanthapon N et al. (2017)
11	Petrochemicals	Hexane, ethanol, methanol, acetone and precious metals like platinum, palladium, rhenium (from the spent catalyst)	Hydrochloric acid	Electrochemical recovery	No chemical addition, high efficiency, possibility for energy and resource recovery	Anode inactivation may happen	Santos PG et al. (2020)
12	Textile	Heavy metals like Cd, As, Pb, Cu, etc. and chlorinated solvents	Acid, reactive and direct dyes using anion exchange resins	Bulk solids and fabrics filtration, nanofiltration, adsorption	Ease of operation, reliable, low power consumption, high efficiency	Expensive regeneration process	Thamaraiselvan C et al. (2018)
13	Metal refinery	Gold, silver, platinum, and other metals like Cd, Mo, Pb, Ni, etc.	Tartaric acid, acetic acid, EDTA	Flotation	Efficient separation, applicable for low grade embedding	Causes environmental pollution, finer grinding particle size is needed	Garole DJ et al. (2018)
14	Solar industry (photovoltaics)	Metals like silicon, silver, copper, aluminium, etc.	Hydrohalic acid	Sedimentation and centrifugation	Labor-intensive, short harvesting times	Less flexibility and suitable for larger volumes	Igoud S et al. (2021)
15	Iron and steel	Manganese, iron, aluminium, silicon, titanium, vanadium, etc.	Sulphuric acid, butyric acid, and other organic and mineral acids	Flocculation and precipitation	Process simplicity and integrated physicochemical technique	Not cost-effective, system controls are required	Wang LP et al. (2019)
16	Semiconductor s	Metalloids such as antimony, selenium, gallium, germanium, etc.	Sodium chloride, poly-ethylene terephthalate (PET)	Electrodialysis	Property of polarity reversal allows to perform in the absence of chemicals	Ion diffusion is non-linear to applied voltage after certain current density	Eng CY et al. (2019)
17	Dairy	Heavy metals like lead, chromium and trace elements like zinc, copper, iron	Citric acid, ammonium molybdate, potassium antimony tartarate, lactic acid, etc.	Diffusion dialysis	Uniformity, optimum performance, low neutralization costs	High operational cost and high consumption of water and energy	Brião V. B et al. (2019)
18	Leather	Synthetic tanning agents such as formaldehyde, glutaraldehyde, phenols, acrylates, etc., sulfonated oils, metals like cerium, manganese, chromium, aluminium	Formic acid, phosphoric acid, nitric acid (which are complexing agents for the removal of chromium from leather scraps)	Distillation and rectification	Energy saving operation, less theoretical stage requirements	High operating costs	China CR et al. (2020)
19	Paper and pulp	Carbon, disulfide methanol, acetone, methanol (used for wood-chips digestion, spent liquor evaporation)	Potassium nitrate, nitric acid, sulphuric acid, saccharinic acid, resin acid, formic acid	Activated carbon adsorption	Provides high surface area and significant stability	Product recovery requires special, expensive distillation/ extraction	Elakkiya E et al. (2020)
20	Oil extraction	Metal halides like stannous chloride and crude oil	Polyacrylic acid,	Reverse osmosis	Separation of dissolved substances, cost-effective	Possibility of fouling since it is a membrane-based technique	Chang H et al. (2019)

## Aspects of wastewater effluent

Wastewater contains numerous contaminants and toxins which are considered as most serious threat to the ecosystem and public health. In general, wastewater is characterized as: (i) Physical: Temperature, turbidity, total suspended solids, color, odor (ii) Chemical: COD, TOC (Total Organic Carbon), heavy metals, Dissolved Oxygen (DO), toxic substances, p^H^, phosphorus, sulfur, chlorides and other trace elements (iii) Biological: BOD, microbes such as bacteria, viruses, parasites, oxygen required for nitrification and microbial population (Zhuang LL et al. 2019) Upon releasing the water containing these impurities into surface, ground and sea water, there occurs nutrient depletion, disorderliness of water quality, and bringing down the DO content which in turn affects the aquatic ecosystem. Feed and produced water quality, and production process mainly determines the feature, quality, composition and volume of the effluent, which in turn directs the cost for effluent disposal and treatment methods. Contaminants of wastewater could be either organic (phenols, aromatic hydrocarbons, pesticides, phenols, etc.), inorganic (nitrogen, phosphorus, sulfur, chlorides, heavy metals like Cd, As, Pb, Hg, Zn, Ag, Ni etc.), radioactive (nuclear material); according to which pre- and post-treatment systems are implemented.

Moreover, other industrial effluents such as manufacture of cement, cannery, metal container, synthetic resins and polymer, soft drinks, soap and detergent, viscous rayon, gelatin, explosives, bleach-liquor, dye, asbestos, chlor-alkali, metal pickling, coffee pulping, slaughter house, meat pickling, etc., contributes in environmental pollution/degradation in addition to the major industrial wastewater. Therefore, effluent treatment plants that comply with the terms of regulations specifying the characteristics of the effluent that is discharged in water stream are in need to secure ecosystem and public health.

## Salient pollutants of concern in industrial effluent and their effects in environment and public health

The major contaminants in wastewater are nitrogen and phosphorus, hydrocarbons, heavy metals and microbes.

### Nitrogen and phosphorus

Ammonia is generally present in wastewater, which is the main form of nitrogen, is known to be toxic. The intake of nitrate containing water could lead to methemoglobinemia, also called as blue babies syndrome in infants and other susceptible individuals (J. Ruiz et al. 2011). Phosphorus is considered as one of the major eutrophic nutrient which have an impact on accelerating the chlorine content required for disinfection of water bodies, which could enhance the increasing risk of cancer and leads to the stimulation of harmful microbes such as *P*. *fisteria* that causes and eye and respiratory irritation.

### Hydrocarbons

The existence of hydrocarbon pollutants in wastewater effluents leads to several environmental and health impacts such as threat to fishery, marine habitats of wildlife, human health, and leads to demolition of ecological balance (Mohammadi L et al. 2020).

### Heavy metals

Heavy metal found in the effluent have a tendency of binding with proteins, thus altering their confirmation and inactivating them, which often results in health complications such as skin irritations, vomiting, nausea, anaemia, disturbing protein metabolism, etc. (Akpor OB et al. 2014). Heavy metals such as zinc, copper, nickel, arsenic, etc., are known for their toxicity, even at extremely low concentrations, due to which, they cause detrimental threat to human health and the flora and fauna of receiving water bodies.

### Microbes

The major pathogenic protozoans present in the industrial wastewater are *Giardia* and *Cryptosporidium* that have the capacity to cause acute and chronic diseases with short- and long-term effects (Dadrasnia Arezoo et al. 2017), such as degenerative heart diseases and stomach ulcers with severity.

Consequently, due to large-scale industrialization and increase in population density, the society is faced with issue regarding wastewater management. The effluent generated due to industrial activities comprises a prime cause of pollution, which is of great concern on water quality management. Moreover, the hazard of non-biodegradable and intractable pollutants in water is their potential to exist in natural ecosystems for a prolonged period and have their ability to accumulate in consecutive levels of biological food chain. Considering the mentioned destructive impacts, variety of treatment processes is required for the wastewater effluent before being discharged into the environment.

## Strategies involved in wastewater treatment

The process of wastewater treatment involves collection of effluent from industries via underground drainage system and subjected to treatment plants wherein, water is put through various treatment levels such as primary, secondary and tertiary. Initially, stages such as screening (unit operation) and odor control are performed prior treatment to prevent stinking and clogging by eliminating materials like plastics, paper, etc. and using chemicals for neutralization. Coarse screens are employed for the removal of large solids and debris, whereas fine screens for removing substances that creates operational and maintenance issues in downstream processing (Bhandari VM et al. 2016).

### Primary treatment

Primary treatment involves separation or removal of precipitable organic and inorganic contaminants from the water by treating them in large tanks for the sedimentation of biotic solid matter at the surface of tanks, which are then, detached using scrappers for grit removal. Treatment units such as settling tanks/clarifiers, septic tanks, anaerobic and UASB (Upflow Anaerobic Sludge Blanket) are integrated in this stage. Additionally, comminutors and grinders are utilized for shrinking the coarse solids so that floating and settle-able solids can be removed during downstream treatment operations (Alvim CB et al. 2020), Currently, detritus tanks are implemented whose fundamental function is to gather utmost quantities of fine particles from the water by enhancing the detention time, whereas organic materials will be existing in the water for subsequent treatment levels (Brown AK et al. 2018). Thus, primary clarification/treatment aims at providing compatible water for the next (secondary treatment) levels.

#### Preliminary treatment: screening—grit removal

Wastewater contains a large amount of solids and grits that can cause damage to the treatment equipment. This can be removed by the process screening. Screen is a device with openings, generally of a uniform size, comprises of coarse suspended and floating solids which are present in a wastewater stream. The screening element comprises of parallel bars, rods or wires, and the openings generally have circular or rectangular slots. The smaller the screen opening, greater the quantity of the screenings (Meerbergen K et al. 2018). Grits are heavy inorganic solids such as sand, metal fragments, egg shells that are heavier than the organic biodegradable solids in the wastewater. Removal of grit prevents unnecessary abrasion and wear of the mechanical equipment. A grit chamber may be horizontal flow or vertical flow and is manually or mechanically cleaned. Grit of a properly designed and operated chamber is free from organic matters which may be used as land fill (Hoiberg B et al. 2021). If grit contains organics in high proportion, it is disposed of by burial or used as manure.

#### Sedimentation

Sedimentation is a treatment process in which suspended particles, like flocs, sand and clay are removed from the wastewater. This process happens naturally when water is still, because gravity will pull the heavier sediments down to form a sludge layer. The advantage of sedimentation is that it minimizes the need for coagulation and flocculation. Additionally, sedimentation can be used after coagulation to increase the effectiveness of ongoing filtration in the process (Chu BT et al. 2018). Sedimentation wastewater treatment requires specialized tanks. A sedimentation tank provides the necessary support to make sure the particles settle. The types of sedimentation tanks used are Horizontal flow tank, Multi-layer tank, Radial flow tank, Floc blanket sedimentation and Settler tank.

In addition, techniques such as centrifugal separation, neutralization, gravity separation, induced/dissolved air flotation, ultrafication, etc. are employed in primary treatment stage.

### Secondary treatment

Secondary treatment process involves the use of microbes (so-called biological treatment) that metabolize the organic matter present in the water and generates inorganic by-products, after which, microbes are eliminated via sedimentation method. This treatment process is categorized into two groups. First, activated growth process or fixed film process that includes contact bed, intermittent filter, trickling bed, rotating biological contactor, according to which, the biomass is made attached to inert medium such as rock, ceramic, plastic, slags etc., for the conversion of organic matter (Raju S et al. 2020). Second, suspended growth processes that embraces activated sludge process, aerated lagoons, oxidation ponds, which involves suspending the microbial cells naturally or mechanically for organic matter conversion (Kumar R et al. 2019). Hence, residual organics and suspended solids from primary treatment, are removed through secondary treatment and thereby making the water consistent for tertiary treatment.

#### Activated sludge

The activated sludge is a biological process which takes place after the primary sedimentation. The wastewater obtained from the primary treatment still consist of some suspended and colloidal solids which is agitated in presence of air the suspended solids form nuclei on which biological life develop and gradually build up to larger solids (Zhang H et al. 2019). Generally, the activated sludge is a brownish floc-like substance consisting of organic matter obtained from the wastewater and inhabited by myriads of bacteria and other forms of biological life. Activated sludge with its living organisms has the property of absorbing or adsorbing colloidal and dissolved organic matter. The biological organisms utilize the absorbed material as food and convert it to inert insoluble solids and new bacterial cells. Much of this conversion is a step-by-step process. Some bacteria attack the original complex substances to produce simpler compounds as their waste products (Sepehri A et al. 2020). Other bacteria use the waste products to produce sill simpler compounds and the process continues until the final waste products can no longer be used as food for bacteria. The activated sludge must be kept in suspension during its period of contact with the wastewater being treated by some method of agitation so that they do not get settled in the bottom.

#### Trickling filter

Trickling filters are used to remove organic matters from the wastewater occurs under aerobic treatment of wastewater. The trickling filter consists of a cylindrical tank and is filled with a high specific surface area material, such as rocks, gravel, shredded PVC bottles, or special pre-formed plastic filter media. A high specific surface provides a large area for biofilm formation. Organisms that grow in the thin biofilm over the surface of the media oxidize the organic load in the wastewater to carbon-dioxide and water, while generating new biomass (Liang Q et al. 2020). The incoming pre-treated wastewater is ‘trickled’ over the filter, e.g., with the use of a rotating sprinkler. In this way, the filter media goes through cycles of being dosed and exposed to air.

Aerated lagoons, contact media, disk contractors, extended aeration, high rate activated sludge are some other methods applied in secondary treatment stage.

### Tertiary treatment

Tertiary treatment of wastewater is required to remove the enduring contaminants/nutrients/pathogens that accomplish over preliminary treatment level, to meet reuse quality standards and to make the treated water appropriate for land applications and direct discharge into water bodies like rivers, lakes, ponds, etc. It involves steps such as nutrients removal, disinfection, ion-exchange, membrane process and filtration. Advanced oxidation process falls under this treatment which aims at components necessary to the toxic activity so as to achieve capacity to influence the discrete toxic effect on the compound. Recent findings reported that tertiary filtration and chlorine disinfection are effective in reducing the emerging environmental contaminants like antibiotic resistance gene in the wastewater effluent.

#### Disinfection (UV/chlorination)

Killing, removal or destruction of microorganisms is generally referred as disinfection. These disinfectants are added in the wastewater treatment to resist against the microorganisms present in the contaminants. These can be done either by chlorine or by UV radiation (Azuma T et al. 2021). Chlorine is generally a greenish-yellow gas while applying a high pressure it gets changed from gas to liquid. Introduction of chlorine to water plays a very effective role for removing almost all pathogenic microorganisms. While, UV or Ultraviolet rays also been used extensively in the tertiary treatment. The UV light causes disinfection by changing the biological components of microorganisms specifically breaking the chemical bonds in DNA, RNA, and proteins and the radiations also control the regrowth potential within the distribution system.

Techniques involving granular/activated carbon, reverse osmosis, electro-dialysis etc. are in existence in tertiary treatment step.

## Different techniques of effluent treatment

Table 9 reports the various methods employed for effluent treatment. Some of the most common treatment methods include membrane filtration, adsorption, photocatalysis, electro-coagulation, bioaugmentation, ozonation.

**Table 9:** Comparison among effluent treatment methods

S.no	Treatment methods	Illustration	Merits	Demerits	References
1	Membrane filtration	Removal of solids from wastewater based on ultrafiltration/microfiltration	Feasible to attain the required water and discharge concentration factors	Not applicable for shear sensitive materials and expensive	Barakat M.A (2011)
2	Activated carbon adsorption	Adsorption of toxic organic compounds from the effluent	Pertinent for the removal of wide variety of dyes like azo, reactive dyes, etc.	Regeneration is expensive and involves adsorbent loss	Barakat M.A (2011)
3	Photocatalysis	Advanced oxidation technology for eliminating the determined organic compounds and microbes from wastewater	Low operational costs and absolute mineralization of chemical substances	Fouling of photo- catalysts	Threrujirapapong T et al. (2017)
4	Electro-coagulation	Destabilization of suspended, emulsified and dissolved contaminants in the effluent by the application of electric current	No chance of secondary pollution as no chemicals are added	Requires regular replacement of anode	Gatsios E et al. (2015)
5	Bio-augmentation	Enzymatic treatment to remove pollutants from the wastewater	Improves contaminant degradation	Rehabilitation result may be incomplete	Bora T et al. (2014)
6	Biodegradation	Primary removal mechanism for emerging organic pollutants in wastewater	Simple, economically feasible	Necessary to create an optimally favorable environment	Barakat M.A (2011)
7	Nanotechnology	Application of nanoparticles for the removal of contaminants from the effluent	Complete degradation of pollutants	Recovery of nanocatalyst is tedious	Bora T et al. (2014)
8	Ozonation	Waste water treatment technique based on the infusion of ozone in water	Increases the dissolved oxygen content in water	Cost of treatment is comparatively high	Cano Quiroz A et al. (2011)

### Membrane filtration

Wastewater containing heavy metals such as copper and cadmium are produced by industries such as textile; to which the technologies like Reverse Osmosis (RO) and Nanofiltration (NF) are applied to remove those heavy metals in the form of ions. Studies have reported that the technique of membrane filtration is capable of removing more than 90% of the copper ion existing in the effluent (Gunatilake SK 2015). This technique entails bonding the metals to a particular bonding agent from the wastewater stream by separation processes for which a hybrid process of flotation and membrane separation is employed by combining the microfiltration modules right away into the flotation rector.

In pharmaceutical industry, membrane filtration accounts almost 9% for the removal of effluents. The purpose is to remove viable and non-viable particles to clarify or sterilize the solution. The size of the pores in filters also responsible for the separation as the screen allows particles of a specific size through but traps molecules that are too large to fit through the pores (McKinnon BT et al. 2018). One of the main advantages of using filters is for testing end product sterility. The rate of flow through a filter is affected by the resistance of the filter, the viscosity of the solution, and pressure which sometimes make them less efficient in the removal of effluents.

### Adsorption

Adsorption by solid adsorbents is one of the potential methods for the removal of dissolved organic contaminants such as dyes, from the industrial wastewater. Adsorption with active carbon is frequently employed as tertiary purification for the elimination of organic micro-pollutants and metals in organic complexes from wastewater (Lakherwal D. 2014). Adsorbents such as zeolites are used for the removal of iron, ammonium, nitrate, manganese or heavy metals from the effluent. The superiorities of this technique are low cost, simple operation, high efficiency and ability to utilize adsorbent materials from different sources.

This process is mainly carried out in textile industry for the removal of the dye from the effluents generated. Particularly, nanoscale materials like hydrogel are great adsorbers of dye materials. The main advantage of using hydrogel for its high flexibility along with its ability to interact with other nanoscale adsorbents particularly for purification process (Van Tran V et al. 2018). But sometimes, due to the infusion of filamentous organisms in gels, chances of contamination of the organisms may occur.

### Photocatalysis

Photocatalysis is a promising method for the treatment of contaminated water. This method makes use of sunlight, thereby it is cost-efficient, eco-friendly, and invariably applicable. Photocatalysis is used to break down a broad variety of organic materials, dyes, crude oils, microbes, inorganic molecules such as nitrous oxide, and in combination with precipitation and filtration, used to remove heavy metals (Mahalingam S et al. 2018). In addition to water purification, this method with nano-based catalysts is used to prevent air pollution and in building materials for self-cleaning surfaces. Titanium oxide (TiO_2_) is the most commonly used photocatalyst for which UV irradiation from sunlight or artificial light is required for its activation.

This method is mainly used in oil manufacturing industry, for the removal of phenolic compounds. TiO_2_ is a semi-conductor photocatalytic used in reduction and removal of these harmful and toxic compounds produced from wastewater. This is highly feasible process for the treatment of both produced waters and waste water runoff from garage forecourts (Chen D et al. 2020). But one of the greatest disadvantages in this process is poor electric adsorption and treatment of highly concentrated organic pollutant.

### Electro-coagulation

Treatment of industrial effluent by electro-coagulation is an efficient method to remove number of pollutants such as organic materials, colorants, minerals and heavy metals. Studies have reported that this technique could be employed for the simultaneous removal of aluminium (from the electrodes) and chromium (in the effluent) accommodated in the effluent (Syam Babu D et al. 2020). This method can eliminate organic and toxic pollutants that are dissolved in wastewater by direct or indirect oxidation. The electrochemical reaction generates strong oxidants such as hypochlorite, hydrogen peroxide on electrolysis, which destroys the pollutants in the effluent.

In tannery industry, electro-coagulation is one of the most efficient technology as well as low operational cost. This provides a direct connection between metal electrode immersed in wastewater. The current causes the dissolution in metal electrode and dissolves metal ions form coagulated particles and metal hydroxides (De La Luz-Pedro A et al. 2019). Sometimes, the electrodes may dissolve into wastewater stream as a result of oxidation, which needed to be changed often.

### Bio-augmentation

The inflating application of enzymatic treatment is generating an increasing demand for biocatalysts that reveals better characteristics. Enzymes remove recalcitrant pollutants by precipitation or transformation to other products by precisely acting on them (Nzila A et al. 2016). Enzymatic processes are considered as clean and green and provide advantages such as rapid, ease of operation and control, and pliability to change in temperature.

Bacillus species is mainly used in agricultural industry, by bio-augmenting the number of microorganisms in the wastewater increases rapidly which in turn able to speed up the biodegradation of the contaminants (Pandey AK et al. 2017). Using microorganisms specifically adapted to digest certain type of contaminants can help make bioaugmentation more successful. While they have its own advantages sometimes due usage of wrong type of bacteria can result in clogging of contaminants.

### Nanotechnology

Nanotechnological processes such as nanofiltration, nanomaterials for catalysis, photocatalysis, water disinfection, adsorption of pollutants, nanoscale zerovalent iron (nZVI) play a vital role in removing various types of contaminants and permits high degree of water purity. Nanotechnology offers a wide range of solutions for membrane materials such as ceramic nanomembranes, polymers with metal oxides, carbon nanotube, zeolites, aquaporin, etc. The three types of nanomaterials that can be regarded as the promising ones are nanoadsorbents, nanomembranes and nanocatalysts. There are certain factors that influence the production of nanomaterials such as:

- Practical opposition on the use of specific nanotechnologies
- Production and operating costs
- Effects on human life and environment

The nanoparticles are mainly involved in the decolorization of textile dyes and the remediation of textile effluents. Numerous types of nanoparticles are used for the purpose and are unique based on their physicochemical properties. Also, nanoparticles are used as adsorbent for the removal of heavy metals are non-toxic and have high adsorption capacity and to adsorb pollutants in less concentration, adsorbed pollutants that can be easily removed from adsorbent surface and can be recycled for numerous times (Palmieri V et al. 2021). The smaller size of the nanoparticles increases the surface area which enhances the chemical activity and adsorption capacity of NPs for the adsorption of metals on their surface. The frequently used NPs for the adsorption of heavy metals are activated carbon, carbon nanotubes, manganese oxide, graphene, zinc oxide, titanium oxide and ferric oxides.

Nanotechnology is used in food packing sector in form of nanosensors or carbon nanotubes are used for rapid detection of microbes present in the food materials. Generally, toxin antibodies are connected with the nanotubes which indeed cause a detectable change in conductivity during the detection of water borne toxins present in them (Enescu D et al. 2019). These tubes are further used for detection of aroma or gases released by the food items by giving signals.

Nanotechnology used in medical field in form of magnetic nanoparticles (magnetic Fe_3_O_4_) as a contrast agent for magnetic resonance imaging, comprehensive in vitro/in vivo toxicity studies have already been carried out (Nikolova M et al. 2020). Through this technology, a bandage has been introduced electrical pulses to a wound using electricity produced by nanogenerators worn by the patient. For trauma patients with internal bleeding another way to reduce the blood loss is needed are developing polymer nanoparticles that act as synthetic platelets. Lab tests have shown that injection of these synthetic platelets significantly reduces blood loss.

### Ozonation

Ozone can be constructively used for the treatment of municipal and industrial wastewater. It is a powerful oxidant and leaves no residual harmful product and no sludge disposal problem and accelerates the DO content of wastewater which aids additionally in the degradation of residual pollutant. Investigations reveal that ozone is effective 25 times more than hypochlorous acid, 2500 times more than hypochlorite, 5000 times more than chloramines; so that this technique finds application in various fields of wastewater treatment (Ledakowicz S et al. 2017). This technique has variety of industrial applications such as paper and dyeing industry effluent treatment for color removal, treatment of toxic and cyanide waste, eradication of heavy metals and phenols from wastewater, deodorization and treatment of gaseous effluent, etc.

Ozonation is mainly used in food industry, mainly for the production of black pepper in substitute of ethylene oxide for the decontamination of whole black peppercorns and ground pepper balls (Upadhyay et al. 2017). Ozone treatment of ground black pepper resulted in slight oxidation of volatile oil constituents but ozone had no significant effect on the volatile oils of whole peppercorns.

Table 10 shows the recent trends/advancements in treating the effluent from various industries. Since conventional methods are found to be expensive and not suitable for small-scale process, employment of hybrid system for treating wastewater effluents overcomes the drawbacks while requiring low capital investment, less labour-intensive and economically feasible and accessible.

**Table 10:** Modern methods of treatment of wastewater effluent from different sources

S.No	Type of industry	Effluent composition	Recent advancements in effluent treatment	Treatment category	Target of removal	Merits	Demerits	References
1	Electric power plants	Methane, siloxanes, carbon-dioxide, ammonia, hydrogen sulphide, suspended solids	Development of microbial fuel cell with biocatalysts for concurrent electricity production and pollutant removal from effluent	Biological	Ammonia, Carbon-dioxide, methane through nitrification, denitrification and bio-mineralization	Offers better aversion to environmental stress	High cost and short life span	Guo Y et al. (2020)
2	Battery manufacture	Metals like aluminium, cobalt, copper, lead, iron, hydrogen fluoride, lithium, manganese and nickel	Application of pyro, hydro and biohydro—metallurgy for metal extraction from the effluent	Mechano-chemical	Nickel, lithium, cobalt	Separation of valuable metals and economically viable	Reliant to chemical composition and high energy consumption	Mossali E et al. (2020)
3	Nuclear power plants	Gaseous (inert gas, halogen, aerosol) and liquid (tritium) radioactive substances	Solidification with barriers to cease water and prevent the water radio-nuclide migration and droning in intense development secluded with biosphere	Chemical	Radioactive materials	Harmless to ecosystem and human beings	High operational cost	Ye et al. (2016)
4	Mines and quarries	Sulphide minerals such as suchlike pyrite (FeS_2_), pyrrhotite (FeS)	Bioremediation and phytoremediation that relies on microbes to degrade the organic contaminants in the wastewater effluent	Biological	Polymetallic sulphides	Economical and less disruptive to the environment	Sensitive to toxicity level	Agboola O et al. (2020)
5	Food	Organic compounds, suspended solids, sugar, fats, color, preservatives and nutrients	Employment of hydrophobic neoteric solvents as extractants such as eutectic solvents, ionic liquids, bio-based solvents etc. for phenolic compound separation from food effluents via liquid–liquid extraction	Physico-chemical	Phenolic compounds (flavonoids and non-flavonoids)	Facilitates the separation of high value-added compounds such as phenolic anti-oxidants	Time consuming process and the solvent should be evaporated to concentrate the extract	Canadas R et al. (2020)
6	Agriculture	Antibiotics, synthetic compounds, organic compounds and suspended solids, nitrogen and phosphorus	Incorporation of micro-algae into wastewater effluent based on autotrophic nitrification and heterotrophic denitrification for intensified biological N & P removal	Biological	Nitrogen, phosphorus and other organic waste	Eco-friendly and sustainable alternative to conventional biological treatment	High energy requirement and overall cost	Mohsenpo ur SF et al. (2020)
7	Dairy	Lactose, fats, whey proteins, chlorides, sulphate, soluble organics, suspended and dissolved solids, BOD, COD	Implementation of unmodified rice husk (by-product of rice milling) as a biosorbent which gets protonated at low p^H^ and thereby capturing the organic materials to the binding sites	Physico-chemical (adsorption)	Organic substances	Easy accessibility of raw materials and cost-effective	Usage of high adsorbent dosage leads to COD loading	Pathak U et al. (2016)
8	Oil extracting mills	Organic carbon, nitrogen, methane, carbon-dioxide, hydrogen sulphide, suspended solids, BOD, COD	Utilization of palm kernel shell for the development of biomass adsorbent through the integration of zeolite and iron oxide for the adsorption of organic pollutants from the effluent	Physico-chemical (adsorption)	Heavy metals, diligent organic/inorganic contaminants	Increased stability and adsorption efficiency, good separation, aids in the conversion of solid waste to useful adsorbent	High pre-production cost	Jun KC et al. (2020)
9	Petroleum and petrochemicals	Dissolved oil, hydrocarbons, gases like H_2_S, CO_2_ and organic acids	Hybrid system using continuous flow intermittent cleaning biofilm technology -based moving bed biofilm reactor and assimilated native microbial association – based continuous stirred tank bioreactor	Biological	COD and total petroleum hydrocarbons	High resistance to toxic effects, increased mass transfer between hydrocarbon and biocatalyst, highly precise	High operational and maintenance costs	Kuyukina MS et al. (2020)
10	Organic chemicals	Crude oil and grease, hydrocarbons, BOD, resins, pesticides, synthetic fibers, organic chemicals (benzene, toluene, phenols, etc.) and heavy metals (chromium, lead, copper etc.)	Integrated treatment involving fixed biofilm bioreactor, two-phase partitioning bioreactor, sequencing batch reactor to remove the toxic pollutants	Physico-chemical, biological	Heavy metals and other inorganic matter	Technologically and economically feasible	Sedimentation is required to prevent clogging, time-consuming	Awaleh MO et al.(2014)
11	Leather	Volatile organic compounds, heavy metals, COD, BOD, dissolved solids, sulphides, calcium/ammonium salts, chromium, H_2_S	Employment of waste tea leaves (dropped out from teashops/residence) for heavy metal removal from the tannery effluent, due to its good biosorption ability	Physico-chemical (adsorption)	Heavy metals like chromium, iron, nickel, lead	Effective, inexpensive, copiously obtainable cheap	Release of soluble carbon content and applicable only for heavy metal removal	Nur-E-Alam et al. (2020)
12	Paper and pulp	Suspended solids, organic matter, chlorinated resin acids, wood extractives, lignin, cellulose, tannins, diterpene alcohols, BOD, COD	Incorporation of fungal consortium (*Nigrospora sp., curvularialunata sp*.) to remove BOD, COD, lignin and bacterial consortium (*actinomycetes sp*.) that generates laccase enzyme to degenerate cellulose and lignin under alkaline environment	Biological	Lignin, cellulose/hemi-cellulose, BOD, COD	Cost effective, ecofriendly	Complexity in micro-biological mechanism, slow process	Ram C et al. (2020)
13	Iron and steel	Oil and grease, phenol, cyanides, ore particles, sulfur compounds and metal ions	Employment of steel slags (containing iron oxide) to remove metallic iron, and steel slag-based induction furnace for chromium removal	Physico-chemical method (adsorption)	Heavy metals	Economically sustainable, reuse of steel waste	Stability problems	Branca TA et al. (2020)
14	Pharmaceutics	Dissolved and suspended solids, COD, organic matter such as alcohol, aromatic compounds, acetone, antibiotics, chlorinated hydrocarbons	Molecularly imprinting technology that employs molecularly imprinted polymers to produce affinity membranes for the removal of antibiotics from water	Physico-chemical method (membrane filtration)	Antibiotic-tetracycline	High selectivity, affinity, stability, easier operation	High utilization of template molecules	Gadipelly C et al. (2014)
			Nanofiltration which is pressure driven membrane separation process for eliminating the antibiotic concentration from the wastewater effluent	Physico-chemical method (membrane filtration)	Antibiotic-amoxicillin	High operational efficiency	Expensive and high energy consumption	
15	Textile	Dyes and fibers (reactive, vat, azoic), toxic chemicals (acids, alkali, surfactant-dispersing agents), heavy metals (copper, chromium, cadmium, zinc etc.)	Photocatalytic degradation using TiO_2_ nanoparticles,	Chemical (photocatalysis)	Dyes	Application of nanotechnology in textile effluent treatment is efficient in eliminating and retrieving pollutants	Costly, instability of nanoparticles	Kumar PS et al. (2017)
			Carbon-based nanomaterials	Physico-chemical (adsorption of pollutants)	Organic/inorganic contaminants			
			Nanosorbents	Physico-chemical (adsorption of pollutants)	Metal oxides			
			Zeolites, carbon nanotubes,	Physico-chemical (adsorption of pollutants)	Heavy metals			

### Stripping

Wastewater with high concentration of ammonia that negatively influences the environment and public health. Mass transfer is the key principle that assists this method, according to which, leachate is allowed to contact with air to strip the ammonia gas present in it. Various parameters such as temperature, p^H^, air to water ratio, concentration of ammonia in the water etc., have impacts on treatment efficacy (Ferraz FM et al. 2013). This method could be combined with principles like cathodic adsorption, sticking probability and absorption for effective removal of ammonia from effluent and thus making it as hybrid technology.

The working mechanism employs a sieve tray air ammonia stripper is employed in which a layer of stainless steel that serves as an attracting plane for the fouling deposits generated. Since stainless steel is cost-effective and has anti-corrosive properties for prolonged exposure time, it is considered as suitable plane for scale deposition. A small beam aperture is incorporated into the stripper via which supersonic molecular beam is collimated directly to the steel surface that enhances the trapping/attracting feature of the plane towards CaCO_3_ and thereby removing the fouling deposits. Absorption process is carried out to collect the ammonia gas from stripper and preventing their release into the atmosphere. On electrolysis, the heavy metals in the form of ions (Cr^3+^, Fe^2+^, Cd^2+^, etc.) gets migrated towards negative electrode (Stainless steel). Therefore, Steel surface plays a dual role as attracting plane and electrode and aids in removing ammonium, organic compounds and heavy metals from the leachate, hence converting it to potable water. This type of integrated method is suitable for agriculture industries.

## Disputes and outlook

Wastewater effluent treatment involves challenges such as energy consumption, labour intensiveness, environmental impression and lower energy efficiency of decentralized treatment process. The recurrent attribute of all the advanced treatment technology is that they stimulate zero-discharge system, which is otherwise known as closed loop system that is delineated to recycle, filter and reuse the water, whose execution is highly nominated to attain sustainable and ecological wastewater treatment, that leads to the reduction of pathogens in surface and ground water to secure the public health. Membrane-based technologies are constructively employed for low-strength effluents which are not appropriate for treatment using anaerobic reactor due to low biogas potential, yet they offer certain drawbacks to overcome like high-energy demand due to fouling, low COD/sulfate ratios, inadequacy of alkalinity, etc. (Sharma A et al. 2020).

## Critical analysis on future scope of the study

It is essential to practice standard, advanced and suitable treatment techniques for industrial wastewater, since it declares a remarkable threat to the health and environment. On that account, this review prospects the plan and design of wastewater treatment plants based on current and future exploration to overcome the issues related to effluent treatment and reuse potential of treated water. Rapidly developing cutting-edge technologies for wastewater treatment such as micro-coagulation, biosorption, microbial fuel cell systems, etc. serve as distinct medium of future substantial methods. In this review, integrated methods for wastewater treatment like phytosorption with adsorption, stripping with absorption; filtration and evaporation with photocatalytic adsorption, sedimentation with immobilization, nanotechnology with bioaugmentation, etc. have been discussed which are effective in capitalizing the inclusive advantages of the approach intricated with enhanced efficiency and reduced operational and maintenance costs. These hybrid treatment technologies through which reusable water is obtained are not limited only to the effluent treatment, however, also for the utilization of solid wastes and by-products from various industries, thus enhancing waste to wealth concept in industrial effluent treatment. While the calamity of potable water is of spreading challenge in the current century, reutilization of treated effluent is necessary for which analytical, hydrodynamic, computational and sophisticated simulation models like fluid dynamics, black-box model, 1-D layer model (clarifying model), Vitasovic’s layer model (thickening model), artificial intelligence, geographic information system, etc. and empirical/statistical models like double exponential model, Takács’ model (settling velocity function), IAWQ activated sludge model (kinetic based), etc. could be possibly used in future research for resolving the issue of water demand. Reclamation of resources such as heavy metals, minerals, water, dyes, fibers, organic matter, volatile compounds, oil, etc. from the wastewater effluent opens the door for novel business strategies and right set of circumstances and also reinforces the core of energy, environment, health and water. Hence, conjugation of techniques like decolorization with aerated biodegradation in addition to the above-mentioned integrations would seem to be a supreme field for future research in industrial effluent treatment.

## Conclusion

It is perceptible that industrial wastewater effluent is obviously loaded with toxic heavy metals, dyes, organic compounds, etc. that are unable to be treated efficiently thereby constituting as a pollutant to the environment. Disposal of imperfectly treated effluents from the industries lead to the humiliation of ecosystem thus making the water inappropriate for daily requirements of life. Effluents released from industries such as textile, paper, pulp and oil, brewery, food and beverages etc., are convinced to provide a comprehensive profile of industrial wastewater and also the disposal problems. This paper is a review of course of actions that could be implemented in treatment process for the recovery and reuse of water; and also inspected the arising problems and technical possibilities to overcome the same related to treatment systems. Although there evolved various traditional and conventional treatment methods for purifying the industrial wastewater, implementation of integrated water reuse design promotes the use of retrieved wastewater that offers adequate workability to assure fidelity in water supply. The prevailing quality of this survey is based on current regulatory environment for wastewater management and recognized to assist the upcoming research attempts in this field. This paper looks over literary texts in a way that it focuses on techniques and their applicability in industries, efficiency, design parameters, and specificity of various industrial effluent treatment plants nevertheless does not include particulars concerning feasibility, experimental data analysis and degradation kinetic parameters/equations of the wastewater treatment design which can be concentrated in future studies.

## Data Availability

All the data mentioned are available from the authors upon reasonable request.
